# Celiac Disease and Autoimmune Thyroid Disease in Children with Type 1 Diabetes Mellitus: Clinical and HLA−Genotyping Results

**DOI:** 10.4274/jcrpe.v2i4.151

**Published:** 2010-11-03

**Authors:** Ayça Törel Ergür, Gönül Öçal, Merih Berberoğlu, Pelin Adıyaman, Zeynep Şıklar, Zehra Aycan, Olcay Evliyaoğlu, Aydan Kansu, Nurten Girgin, Arzu Ensari

**Affiliations:** 1 Department of Pediatric Endocrinology, Faculty of Medicine, Ufuk University, Ankara, Turkey; 2 Department of Pediatric Endocrinology, Faculty of Medicine, Ankara University, Ankara, Turkey; 3 Specialist Dr, Department of Pediatric Endocrinology, Sami Ulus Hospital, Ankara, Turkey; 4 Department of Pediatric Endocrinology, Cerrahpaşa Faculty of Medicine, Istanbul University, Turkey; 5 Department of Pediatric Gastroenterology, Faculty of Medicine, Ankara University, Ankara, Turkey; 6 Department of Pathology, Faculty of Medicine, Ankara University, Ankara, Turkey; +90 312 204 41 70+90 533 691 76 28aycaergur@superonline.comBilkent 1, Çamlık Sitesi, D: 2/11 Bilkent, Ankara, Turkey

**Keywords:** type 1 diabetes mellitus, autoimmune thyroiditis, celiac disease

## Abstract

**Objective**: Increased prevalence of celiac disease (CD) and autoimmune thyroid disorders (ATD) in patients with Type 1 diabetes mellitus (T1D) has been widely reported. Such an association may lead to adverse effects on the growth, bone metabolism and fertility, and response to therapy may become difficult. The aim of this study was to evaluate the clinical findings and HLA typing results in patients with T1D associated with CD or ATD.

**Methods**: The association of CD and ATD was evaluated in 38 children with T1D aged 1.5−16.8 years who had been followed for 48.3±28 months. Diagnosis of CD was based on positivity for serum endomysial IgA antibody and histopathological findings of intestinal biopsy specimens. Thyroid autoimmunity was assessed by antithyroglobulin and antithyroid peroxidase antibodies and with diagnostic ultrasonographic findings.

**Results**: ATD was detected in 31.5%, and CD−in 7.8% of T1D patients. Subjects with CD showed either no symptoms or suggestive problems such as short stature, hepatosteatosis, pubertal delay and difficulties in the control of diabetes. Patients with ATD had no clinical symptoms. DQ8 was the most prominent finding in CD.

**Conclusions**: It is essential that patients with T1D, regardless of presence or absence of symptoms, should be investigated for CD and ATD.

**Conflict of interest:**None declared.

## INTRODUCTION

Patients with type 1 diabetes mellitus (T1D) are at a great risk for developing autoimmune diseases. It is well recognized that T1D can be associated with celiac disease (CD) and autoimmune thyroid disorders (ATD). Recent studies regarding CD and T1D have indicated that the frequency of this association can vary from 1.7% to 16% (1, 2). The frequency of ATD in patients with T1D is reported to vary from 3.9% to 40% in different populations (3). On the other hand, the frequency of ATD in patients with CD varies from 4.1% t 14% (4). Growth, bone metabolism and fertility can be affected by these autoimmune associations (4). In this study, the aim was to investigate the prevalence of CD and ATD in Turkish pediatric patients with T1D and to correlate the clinical findings and HLA−genotyping results with the above−mentioned autoimmune disorders.

## METHODS

The study group consisted of 38 children (19 boys, 19 girls) with T1D aged from 1.5 to 16.8 years (mean age; 9.4±2.9 years) who had been followed up in our department for a mean period of 48.3±28 months.

The diagnosis of T1D was based on clinical findings (polyuria, polydipsia, polyphagia and weight loss) and presence of hyperglycemia (randomised glucose level ≥200 mg/dL). Pancreatic autoantibodies [Islet cell autoantibodies (ICA), glutamic acid decarboxylase antibodies (antiGAD) and anti−insulin autoantibodies (AIA)] were also evaluated in all children in the study group (5). In addition, HLA−genotyping by polymerase chain reaction was performed in all patients (6). Pancreas−related autoantibodies (ICA, anti GAD, AIA) were determined using radioimmunoassay (RIA) methods (7, 8, 9).

The immunoglobulin A (IgA) antiendomysium antibody (EMA) test was selected as the screening test for CD and performed in all patients. IgA deficiency was excluded in each patient. Serum samples were analyzed for EMA by the indirect immunofluorescence method (10). Intestinal biopsy was performed in patients showing EMA positivity. EMA−positive patients with no clinical symptoms suggestive of CD, but showing typical histopathological findings consistent with CD (villous atrophy, elongated crypts, infiltration of plasma cells, lymphocytes, eosinophils and basophils in the lamina propria), were accepted as silent CD cases, while patients with no clinical symptoms but having intraepithelial lymphocytosis in the small bowel biopsy were considered as latent CD cases. Those who exhibited gastrointestinal symptoms were categorized as classic CD patients, and those who had extraintestinal findings−as atypical CD patients (11, 12). Antibodies for CD and ATD were searched for on admission in all patients. Antibody measurements were rechecked annually. Because variable nutrient absorption due to CD−associated intestinal injury may destabilize diabetic control (13), in patients with metabolic dysregulation, CD was reinvestigated within a period shorter than a year. In patients with CD, after gluten−free diet, the metabolic control was evaluated.

Serum free triiodothyronine (T3), free thyroxine (T4), thyrotropin (TSH), antithyroglobulin (antiTG), antithyroid peroxidase antibody (antiTPO) were measured in all patients. Serum free T3 and free T4 levels were measured by competitive immunoassay method using immunodiagnostic products (14). Serum TSH levels were measured by immunometric method (15). AntiTG and antiTPO were measured by immunometric assay, using immulate 2000 (16). Values above 35 U/mL for AntiTg and 40 U/mL for antiTPO were considered to be positive (16). The thyroid gland was assessed by palpation and graded according to the goitre classification system proposed by the World Health Organization (17). Thyroid sonography was performed by high−resolution ultrasound, using 7.5 MHz probes in each patient. Thyroid volumes were calculated by reference criteria (18). Accordingly, thyroid volumes above 97th percentile were accepted as goitre (18).

## RESULTS

Somatic growth was within normal limits in all patients. The clinical and laboratory characteristics of the patients are shown in Table 1. AIA was the most frequent antibody type at the time of diagnosis of T1D. AntiTPO and antiTG antibodies were present in 29% and 23% of patients, respectively. Twelve of 38 (31.5%) T1D patients were positive for one or two antithyroid antibodies. HLAgenotyping showed that DQ8 was the most frequent type, followed by DQ2. CD was diagnosed in three children among these 38 T1D patients (7.8 %). The general characteristics of cases with T1D and CD are shown in Table 2. In patient 1, who was diagnosed with latent autoimmune diabetes in children (LADC) (19), three of the pancreas−related antibodies were positive. One patient diagnosed with CD had short stature, hepatosteatosis and pubertal delay. The third child had uncontrolled hyperglycemia despite strict insulin and diet therapy. Patients 1 and 2 diagnosed with CD had also ATD. Patients with positive results were subjected to a gluten−free diet, after which, an improvement in the metabolic status was observed only in the third child.

**Table 1:**
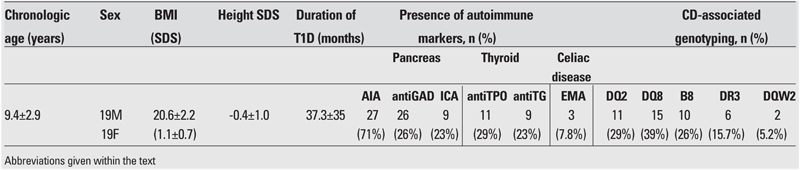
Clinical and laboratory findings of the patients (n=38) (mean±SD or % values given)

**Table 2:**
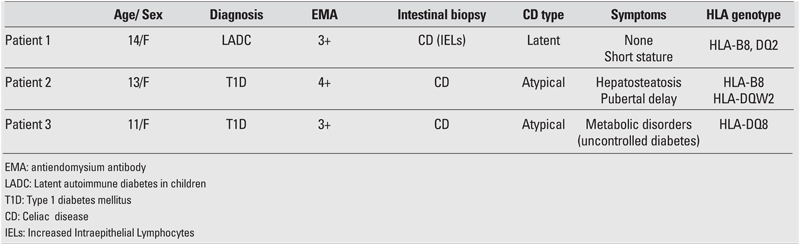
Characteristics of patients with T1D and CD

## DISCUSSION

Autoimmune disorders such as ATD and CD are relatively common in diabetic children and serological screening studies evaluating the prevalence of CD in patients with T1D have gained momentum in recent years. Rozsai et al (10) reported EMA positivity of 6.6% in 196 T1D patients. Among these, 1.5% were symptomatic CD cases. The highest association rate (16.4%) was reported by Barera et al (20). These authors used antigliadin antibody and EMA tests as screening methods (21). Cherubini et al (22) reported EMA positivity in 180 cases with T1D and their 116 healthy male siblings as 6.6% and 5.2%, respectively, emphasizing the need for serological screening for CD in siblings of T1D patients.

In our study, the association rate for CD in T1D patients was 7.8%, a rate which conforms to reported data. One of these three patients was completely asymptomatic, one demonstrated extraintestinal symptoms such as short stature and pubertal delay, and the third had uncontrolled diabetes.

Investigations have been focused on the effect of administering a gluten−free diet based on a diagnosis of CD on the metabolic control of diabetes (23). A diet initiated upon determination of CD in a child with T1D and suffering from malnutrition is expected to lead to weight gain and reduction in the number of hypoglycemic episodes. A decrease in hypoglycemic attacks in pediatric cases with T1D associated with CD after starting a gluten−free diet had been observed by several investigators (21). However, there are also studies reporting no change in the incidence of hypoglycemia and ketoacidosis by gluten−free diet in children with T1D (24). We found improvement in the metabolic control in only one CD patients.

The DR3/DQ2 tissue type determined in one of our patients (Table 2) favors the co−existence of LADC and CD (19). It has been reported that the CD−related antibodies increase in frequency in the first−degree relatives of T1D patients (25). Studies have clearly shown that there is a significantly higher incidence of HLA B8, DR3 and DQW2 in CD. The common genetic background may play a role in the immune response mechanism (1). Recent studies have shown that HLADQ polymorphisms (HLA−DQA1 DQB1) significantly modify the risk of ATD in children with T1D (3). In our study, 39% T1D patients had DQ8 and 29% had DQ2 genotypes that are known as risk factors in CD.

Many patients with T1D are euthyroid at the time of diagnosis of ATD. However, overt or subclinical hypothyroidism was reported in 17−58 % of diabetic patients with positive thyroid autoantibodies (26). In our study, two cases had subclinical hypothyroidism and ten cases were euthyroid.

In conclusion, as also proposed in the literature, we suggest that patients with T1D should be investigated annually for antibodies related to CD and ATD, regardless of presence or absence of symptoms. The number of patients in this study is inadequate to draw a conclusion on the association of HLA genotyping and autoimmune disorders; however, it should be kept in mind that certain HLA groups are prone to autoimmune disorders.

**Table 2:**
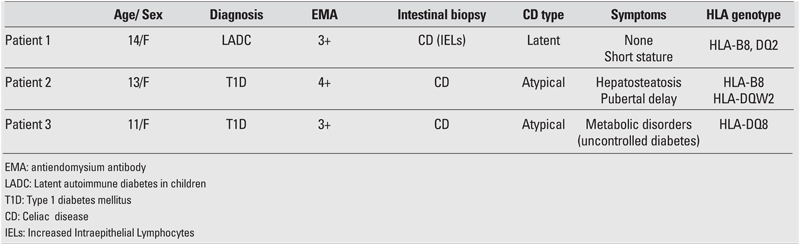
Characteristics of patients with T1D and CD

## References

[1] Lughetti L, Bulgarelli S, Forese S (2003). Endocrine aspects of coeliac disease.. JPEM.

[2] Komer A, Arato A, Madaasy L, Rideg O (2002). Increased risk of other autoimmune disorders in children with type I diabetes mellitus.. JPEM.

[3] Sumnik Z, Drevinek P, Snajderova M, Kolouskova S, Sedlâkovâ P, Pechova M, Vavrinec J, Cinek O (2003). HLA−DQ Polymorphism Modify the Risk of Thyroid Autoimmunity in children with Type 1 Diabetes Mellitus.. JPEM.

[4] M Hakanen, K Luotola, J Salmi, P Laippala, K Kaukinen, P Collin (2001). Clinical and subclinical autoimmune thyroid disease in adult celiac disease.. Dig Dis Sci.

[5] World Health Organization (1999). World Health Organization Consultation Definition, Diagnosis and Classification of Diabetes Mellitus and its complications Part 1. Diagnosis and classification of DM, Report of WHO Consultation−Geneva..

[6] Olerup O, Aldener A, Fogdell A (1993). HLA−DQB1 and DQA, typing by PCR amplification with seqence specific primers(PCR−SSP) in two hours.. Tissue Antigens.

[7] Schatz D, Krischer J, Horne G, Riley W, Spillar R, Silverstein J, Winter W, Muir A, Derovanesian D, Shah S (1994). Islet cell autoantibodies predict IDDM in U.S. shoolage children as powerfully as in unaffected relatives.. J Clin Invest.

[8] Bjork E, Kampe O, Andersson A, Karlsson FA (1992). Expression of the 64 K/ glutamic acid decarboxylase rat islet autoantigen is influenced by the rate of insulin secretion.. Diabetologia.

[9] Vardi P, Dib SA, Tuttleman M, Connelly JE, Grinbergs M, Radizabeh A, Riley WJ, Maclaren NK, Eisenbarth GS, Soeldner JS (1987). Competitive insulin autoantibody RIA. Prospective evaluation of subjects at high risk for development of type 1 diabetes mellitus.. Diabetes.

[10] Rozsai B, Kozari A, Hermann R, Soltesz G (2002). Associated autoimmunity in Type I Diabetes.. JPEM.

[11] Hill ID, Bhatnagar S, Cameron DJ, De Rosa S, Maki M, Russell GJ, Troncone R (2002). Celiac disease: Working Group Report of the First World Congress of Pediatric Gastroenterology, Hepatology, and Nutrition.. J Pediatr Gastroenterol Nutr.

[12] Holmes GK (2001). Coeliac disease and Type 1 diabetes mellitus - the case for screening.. Diabet Med.

[13] Simmons JH, Klingensmith GJ, McFann K, Rewers M, Taylor J, Emery LM, Taki I, Vanyi S, Liu E, Hoffenberg EJ (2007). Impact of celiac autoimmunity on children with type 1 diabetes.. J Pediatr.

[14] Keefer J (1996). Preanalytic considerations in testing thyroid function.. Clinical Chemistry.

[15] Czarnocka B, Ruf J, Ferrand M, Lissitzky S, Carayon P (1986). Interaction of highly purified thyroid peroxidase with antimicrosomal antibodies in autoimmune thyroid diseases.. J Endocrinol Invest.

[16] Kabelitz M, Liesenkotter KP, Stach B, Willgerodt H, Stablein W, Singendonk W, Jager−Roman E, Litzenborger H, Ehnert B, Gruters A (2003). The prevalence of antithyroid peroxidase antibodies autoimmune thyroiditis in children and adolescents in an iodine replete area.. Eur J Endocrinol.

[17] WHO Bulletin (1997). Recommended normative values for thyroid volume in children aged 6-15 years. World Health Organization & International Council for Control of Iodine Deficiency Disorders.. Bull World Health Organ.

[18] Neu A, Ranke MB (1992). Sonographic size of endocrine tissue, in functional endocrinologic diagnostic in children and adolescents.

[19] Aycan Z, Berberoğlu M, Adıyaman P, Ergur AT, Ensari A, Evliyaoğlu O, Şıklar Z, Ocal G (2004). Latent autoimmune diabetes mellitus in children (LADC) with autoimmune thyroiditis and celiac disease.. JPEM.

[20] Barera G, Bonfanti R, Viscardi M (2002). Occurence of CD after onset of type I diabetes: a 6 year prospective longitudinal study.. Pediatrics.

[21] Collin P, Kaukinen K, Valimaki M, Salmi J (2002). Endocrinological Disorders and Celiac Disease.. Endocrine Reviews.

[22] Cherubini V, Fabiani PE, Scalari PA (2002). High prevalance of Celiac Disease in siblings of Type 1 Diabetic Children.. JPEM.

[23] Thain ME, Hamilton JR, Erlich RM (1974). Co−existence of diabetes mellitus and celiac disease.. J Pediatr.

[24] Sumnik Z, Schober E, Waldhor T (2002). Coeliac disease in children and adolescents with type I diabetes mellitus− a multicenter case control study.. JPEM.

[25] Soukonnen T, Iıonen J, Akerblom HK, Savilahti E (2001). Prevalence of celiac disease in siblings of patients with type I diabetes is related to the prevalence of DQB1*O2 allele.. Diabetologia.

[26] Fernândez CM, Molina A, López JL, Gómez JM, Soler J (1999). Clinical presentation and early course of type 1 diabetes with and without thyroid autoimmunity.. Diabetes Care.

